# Compensating for literature annotation bias when predicting novel drug-disease relationships through Medical Subject Heading Over-representation Profile (MeSHOP) similarity

**DOI:** 10.1186/1755-8794-6-S2-S3

**Published:** 2013-05-07

**Authors:** Warren A Cheung, BF Francis Ouellette, Wyeth W Wasserman

**Affiliations:** 1 Centre for Molecular Medicine and Therapeutics at the Child and Family Research Institute, University of British Columbia, Vancouver, BC, Canada; 2 Bioinformatics Graduate Program, University of British Columbia, Vancouver, BC, Canada; 3 Ontario Institute for Cancer Research, Toronto, ON, Canada; 4 Department of Cells and Systems Biology, University of Toronto, Toronto, ON, Canada; 5 Department of Medical Genetics, University of British Columbia, Vancouver, BC, Canada

## Abstract

**Background:**

Using annotations to the articles in MEDLINE^®^/PubMed^®^, over six thousand chemical compounds with pharmacological actions have been tracked since 1996. Medical Subject Heading Over-representation Profiles (MeSHOPs) quantitatively leverage the literature associated with biological entities such as diseases or drugs, providing the opportunity to reposition known compounds towards novel disease applications.

**Methods:**

A MeSHOP is constructed by counting the number of times each medical subject term is assigned to an entity-related research publication in the MEDLINE database and calculating the significance of the count by comparing against the count of the term in a background set of publications. Based on the expectation that drugs suitable for treatment of a disease (or disease symptom) will have similar annotation properties to the disease, we successfully predict drug-disease associations by comparing MeSHOPs of diseases and drugs.

**Results:**

The MeSHOP comparison approach delivers an 11% improvement over bibliometric baselines. However, novel drug-disease associations are observed to be biased towards drugs and diseases with more publications. To account for the annotation biases, a correction procedure is introduced and evaluated.

**Conclusions:**

By explicitly accounting for the annotation bias, unexpectedly similar drug-disease pairs are highlighted as candidates for drug repositioning research. MeSHOPs are shown to provide a literature-supported perspective for discovery of new links between drugs and diseases based on pre-existing knowledge.

## Introduction

Using previously studied and approved pharmaceutical compounds and applying them towards novel diseases or phenotypes - so-called 'drug repositioning' - has emerged as a key issue in biomedical research [1,2]. The cost of developing a new chemical or molecular entity with proven therapeutic benefit and established safety was estimated at over $1.8 billion in 2010, and continues to rise rapidly [3]. Therefore, using compounds with known biochemical mechanism of action and an established safety record for new purposes is an alternative to the high cost of *de novo *compound research [4]. Advances in drug repositioning research have identified potential treatments for Crohn's disease [5,6], and have raised hopes for advances in the treatment of rare, orphan disorders [7].

Informatics-based approaches to drug repositioning are exemplified by the identification of known drug targets in genes arising in genome-wide association studies [8], the prediction of structural suitability of a known compound for a new protein target [9,10], systems biology using gene expression patterns [6,11], and the study of side effects [12]. Underlying many of these informatics approaches has been the availability of reference databases containing information about the relationship between genes, drugs and diseases, such as DrugBank [13], Pharmacogenomics Knowledge Base [14,15], and the Comparative Toxicogenomics Database [16]. The broader informatics approaches to drug repositioning have been recently reviewed [2]. Advances in literature and text analysis methods offer a promising path to drug repositioning based on established knowledge. Text analysis methods have addressed the study of FDA package inserts in the SIDER database [17] to identify side effects, for the comparison of word utilization between drug and disease-related abstracts [18,19], and for the analysis of similarity between gene ontology process annotations assigned to a known drug target and genes in disease-associated pathways. Literature-based drug repositioning has been reviewed [20,21].

The foundation of any text-based analysis is an organized resource of the primary research literature describing the properties contained in the text. The central information source for biomedical literature is the MEDLINE^®^/PubMed^® ^database encompassing over 20 million indexed articles in 2012. PubMed provides a citation resource tailored to biomedical researchers, globally accessible at no charge. This comprehensive database of medically relevant citations is curated by expert annotators at the National Library of Medicine. Each article is indexed with topics from the controlled vocabulary of Medical Subject Headings (MeSH) [22] by domain experts at the National Library of Medicine. MeSH terms include medically relevant categories such as Anatomy, Disease, Chemical Compounds (including pharmacologic compounds) and Psychiatric Disorders. In addition to the topics in the main MeSH hierarchy, additional chemical compounds are indexed through the Supplementary MeSH vocabulary.

Despite the increasing wealth of raw literature knowledge, having means to evaluate and navigate the entirety of this knowledge becomes progressively more challenging. We previously introduced Medical Subject Heading Over-Representation Profiles (MeSHOPs) as a convenient quantitative representation of the properties enriched in a bibliography of scientific literature from MEDLINE [23]. MeSHOPs succinctly describe the most highly associated MeSH terms for an entity of interest. The quantitative comparison of MeSHOPs is shown here to allow the predictive inference of entity-entity relationships in a study of relationships between drugs and diseases. However, we observe that the magnitude of research literature introduces a strong bias into the study of entity-entity relationships, with the most popular diseases more likely to be linked to drugs in the future, and vice-versa. This bias parallels the effect seen when predicting gene-disease relationships via MeSHOPs, where the most popular genes are more likely to be linked to diseases, and vice-versa [24]. It is important to be aware of biases and trends in research that may influence the results of text analysis, and to correct for these biases to better direct research efforts [25,26].

In this report, we investigate the capacity of MeSHOP comparisons to detect functional relationships between pharmaceutical compounds and diseases with an emphasis on the ranking of candidates for drug repositioning research. We demonstrate that MeSHOPs capture the properties of drugs, and that such information can be compared to disease MeSHOPs to reveal functionally relevant relationships. Entities with limited associated literature, such as some rare diseases, are shown to have disproportionate scores in initial MeSHOP comparisons. To account for existing annotation levels of drug and disease entities and identify MeSHOP similarity, we measure the annotation strength for drug and disease entities and incorporate this prior information into the scoring of prediction strength. Using this improved comparison metric we demonstrate that drug and disease MeSHOP comparisons are improved, as validated by the identification of novel associations observed in future publications and against a curated reference collection.

## Methods

### Pharmacological substances

In this paper, we examine the set of drugs, defined as all chemical compounds annotated as having a Pharmacologic Action, taken from both the Medical Subject Headings (MeSH) and Supplemental MeSH vocabularies. Since 1996, indexers at the National Library of Medicine track articles where the action of a drug is discussed (MeSH Basics - http://www.nlm.nih.gov/bsd/disted/meshtutorial/pharmacologicalactionterms/). In 2003, the MeSH Category "Pharmacologic Action" was created, in order to delineate chemical compounds which are used therapeutically as pharmacologic agents. Such annotations are conservatively assigned, requiring a minimum of 20 supporting research articles. We analyze these 6512 drugs with respect to the diseases in the MeSH hierarchy.

### Constructing drug and disease MeSHOPs

The construction of MeSHOPs has been previously described in detail [23], but we provide a description here for the reader's convenience. A MeSHOP is a quantitative representation of the MeSH annotations associated with a set of articles, where the unifying property of the articles is that each addresses the same, specific entity (for example, all articles discussing the entity "Acetaminophen"). Each article has a curator-assigned set of MeSH terms available in MEDLINE. Comparing the observed frequency of each MeSH term annotated to the set of articles relative to the background rate for each term returns a measure of over-representation (see below for additional details). A MeSHOP is a vector of tuples < (*t_1_, m_1_*), (*t_2_, m_2_*), ... (*t_n_, m_n_*) >. For each tuple (*t_i_, m_i_*) in a MeSHOP, *t_i _*is a distinct MeSH term in the MeSH vocabulary and *m_i _*is the over-representation measure for the term *t_i _*. To account for the tree structure of MeSH, for each MeSH term associated with an article, the article is considered associated to all of the parent terms of that MeSH term.

We consider the 6 512 pharmacologic compounds identified in MeSH 2007 as the drug entities. The 4 229 terms in MeSH 2007 in Category C "Diseases" composed the set of disease entities. We take as the set of articles for a specific entity all the MEDLINE articles annotated by the associated MeSH term. These MeSH annotations are manually curated by domain experts at the National Library of Medicine.

### Predicting drug-disease associations

A drug and a responsive disease are anticipated to share common literature annotations, such as metabolic pathways, cellular processes and symptoms, even if no links between the drug and the disease have been previously reported in the literature. To infer novel relationships between a drug and a disease, we perform quantitative pairwise comparisons of MeSHOPs between members of each class. We hypothesize that a previously unassociated disease *t *is likely to be associated with a drug *d *if the MeSHOP *P_t _*for the disease *t *is highly similar to the drug's MeSHOP *P_d _*. When many biomedical terms are common between two profiles, the likelihood for a future association between the entities profiled is expected to increase.

Sixteen distinct similarity measures were evaluated using Receiver Operating Characteristic Area Under the Curve (ROC AUC) scores, from counting measures such as term overlap and term coverage to calculated measures such as Euclidean (L_2_) and cosine distance of p-value profiles (See Table 1). The scores evaluate the shared characteristics from both the drug and the disease MeSHOPs to make predictions. Two baselines are presented for comparison: the number of terms in the drug MeSHOP, and the number of terms in the disease MeSHOP. These baselines consider only the drug MeSHOP alone, or the disease MeSHOP alone, respectively, not using any information from the other MeSHOP.

**Table 1:** Explanation of the scoring functions evaluated.

Scoring Method	Description
Cosine Distance of Term Frequency-Inverse Document Frequency	$\underset{j\in M}{\sum }\left(c_{i}\left(j\right)d_{i}\left(j\right)\right)/\left(\sqrt{\underset{j\in M}{\sum }{\left(c_{i}\left(j\right)\right)}^{2}}\sqrt{\underset{j\in M}{\sum }{\left(d_{i}\left(j\right)\right)}^{2}}\right))$

Cosine Distance of p-values	$\underset{i\in M}{\sum }\left(c_{p}\left(i\right)d_{p}\left(i\right)\right)/\left(\sqrt{\underset{i\in M}{\sum }{\left(c_{p}\left(i\right)\right)}^{2}}\sqrt{\underset{i\in M}{\sum }{\left(d_{p}\left(i\right)\right)}^{2}}\right))$

Cosine Distance of term fractions	$\underset{i\in M}{\sum }\left(c_{f}\left(i\right)d_{f}\left(i\right)\right)/\left(\sqrt{\underset{i\in M}{\sum }{\left(c_{f}\left(i\right)\right)}^{2}}\sqrt{\underset{i\in M}{\sum }{\left(d_{f}\left(i\right)\right)}^{2}}\right))$

Sum of the log of combined p-values	$\underset{i\in M}{\sum }\text{log}\left(c_{p}\left(i\right)+d_{p}\left(i\right)-c_{p}\left(i\right)d_{p}\left(i\right)\right)$

Sum of the differences of log p values	$\underset{i\in M}{\sum }\left|\text{log}\left(\frac{c_{p}\left(i\right)}{d_{p}\left(i\right)}\right)\right|=\underset{i\in M}{\sum }\left|\text{log}\left(c_{p}\left(i\right)\right)-\text{log}\left(d_{p}\left(i\right)\right)\right|$

L_2 _of log-p of overlapping terms only	$\sqrt{\underset{i\in \left(C\cap D\right)}{\sum }{\left(\text{log}\left(c_{p}\left(i\right)\right)-\text{log}\left(d_{p}\left(i\right)\right)\right)}^{2}}$

L_2 _of term fractions of overlapping terms only	$\sqrt{\underset{i\in \left(C\cap D\right)}{\sum }{\left(c_{f}\left(i\right)-d_{f}\left(i\right)\right)}^{2}}$

L_2 _of log of p-values	$\sqrt{\underset{i\in M}{\sum }{\left(\text{log}\left(\frac{c_{p}\left(i\right)}{d_{p}\left(i\right)}\right)\right)}^{2}}=\sqrt{\underset{i\in M}{\sum }{\left(\text{log}\left(c_{p}\left(i\right)\right)-\text{log}\left(d_{p}\left(i\right)\right)\right)}^{2}}$

L_2 _of p-values	$\sqrt{\underset{i\in M}{\sum }{\left(c_{p}\left(i\right)-d_{p}\left(i\right)\right)}^{2}}$

L_2 _of term fractions	$\sqrt{\underset{i\in M}{\sum }{\left(c_{f}\left(i\right)-d_{f}\left(i\right)\right)}^{2}}$

L_2 _of term frequency	$\sqrt{\underset{i\in M}{\sum }{\left(c\left(i\right)-d\left(i\right)\right)}^{2}}$

Term Coverage	$\left|C\cup D\right|$

Term Overlap	$\left|C\cap D\right|$

Number of Drug MeSH Terms	$\left|C\right|$

Number of Disease MeSH Terms	$\left|D\right|$

*M *refers to the set of all MeSH terms, *C *and *D *refer to the MeSH terms for the drug and disease profile respectively. *c(i)*, *c_f_(i), c_p_(i) *and *c_i_(i) *refer to the frequency, term fraction, hypergeometric p-value and term frequency-inverse document frequency for the MeSH term *i *of the drug profile. *d(i)*, *d_f_(i)*, *d_p_(i) *and *d_i_(i) *refer to the frequency, term fraction, hypergeometric p-value and term frequency-inverse document frequency for the MeSH term *i *of the disease profile.

After implementing and evaluating the scoring metrics using AUC scores, a consistently effective metric was determined to be the Euclidean distance of the log of the p-value for the overlapping terms between the drug and the disease. P-values were reported by Fisher's Exact Test based on a hypergeometric distribution of term utilization across a background set of articles. For this report, two background sets are considered. When working within a specific class of entities (e.g. drugs), the background is most appropriately all articles that are associated with one or more members of the entity class. For comparisons between entity classes, a universal background is used. For this study, the universal set contained 17 million MEDLINE articles assigned MeSH terms in MEDLINE 2007.

### Correcting for pre-existing literature annotation

Given the significant impact of annotation bias on pairwise MeSHOP comparison, we introduce a correction of our similarity scores for these pre-existing literature effects. This correction aims to normalize the scores with respect to existing literature annotation, correcting for inherent biases in the scoring methods and revealing associations that are due to the similarity of annotation rather than the amount of annotation (the research "popularity" of the entity).

Expressed formally, let us consider drug-disease relationships, with scores *X_s_*, drug annotation levels *X_c _*and disease annotation levels *X_d_*, where the annotation level is the number of MeSH terms annotated to articles in MEDLINE for the drug or disease. For a given drug *c *and disease *d *with drug annotation level *x_c _*and disease annotation level *x_d _*and a drug-disease score *x_s_*, we want to determine the probability that *x_s _*is more extreme than a random drug-disease relationship score with drug annotation level *x_c _*and disease annotation level *x_d _*:

$$P\left(X_{s}>x_{s}|\left(X_{c}=x_{c}\right)\wedge \left(X_{d}=x_{d}\right)\right)$$

However, this probability can only be directly computed when the set of drugs and diseases is sufficiently large that there are many drugs and many diseases with the same level of annotation. In order to correct for the previously observed bias, we will seek to adjust the significance based on the local distribution of scores observed for similarly annotated entities.

$$P\left(X_{s}>x_{s}|\left(X_{c}\approx x_{c}\right)\wedge \left(X_{d}\approx x_{d}\right)\right)$$

This can be computed by incorporating the properties of conditional probability as

$$P\left(X_{s}>x_{s}|\left(X_{c}\approx x_{c}\right)\wedge \left(X_{d}\approx x_{d}\right)\right)=\frac{P\left(\left(X_{s}>x_{s}\right)\wedge \left(X_{c}\approx x_{c}\right)\wedge \left(X_{d}\approx x_{d}\right)\right)}{P\left(\left(X_{c}\approx x_{c}\right)\wedge \left(X_{d}\approx x_{d}\right)\right)}$$

As well since $P\left(X_{c}\approx x_{c}\right)$ and $P\left(X_{d}\approx x_{d}\right)$ are independent, this can be further simplified to

$$P\left(X_{s}>x_{s}|\left(X_{c}\approx x_{c}\right)\wedge \left(X_{d}\approx x_{d}\right)\right)=\frac{P\left(\left(X_{s}>x_{s}\right)\wedge \left(X_{c}\approx x_{c}\right)\wedge \left(X_{d}\approx x_{d}\right)\right)}{P\left(\left(X_{c}\approx x_{c}\right)P\left(X_{d}\approx x_{d}\right)\right)}$$

We select $P\left(X_{c}\approx x_{c}\right)=P\left(X_{d}\approx x_{d}\right)=0.1$, and compare against the 10% of the drugs that are most similar, annotation level-wise, to the drugs in the relationship of interest, and likewise for 10% of the diseases. Specifically, we take the drugs within ±5 percentile of annotated term counts, and likewise the diseases within ±5 percentile of annotated term counts. The similarity scores for each possible drug-disease pairing between these selected groups are extracted. By comparison against these scores, an empirical significance score of the candidate drug-disease pairing is assigned. Given the 4 229 diseases and 6 512 drugs, selecting 10% yields hundreds of drug and disease peers, and several hundred thousand scores with which to compare.

$P\left(\left(X_{s}>x_{s}\right)\wedge \left(X_{c}\approx x_{c}\right)\wedge \left(X_{d}\approx x_{d}\right)\right)$ is computed by dividing the number of drug-disease relationships with score greater than *x_s _*and with drug and disease annotation similar to *x_c _*and *x_d _*respectively, by the total number of drug-disease relationships. The correction described allows us to separate the effect of the level of annotation for the drug and disease from the similarity of the concepts and allows the user to distinguish high-scoring drug-disease relationships that are primarily due to the annotation level of the drug or disease concept, from high-scoring relationships that arise due to sharing significant profile similarity.

### Validating drug-disease associations

To evaluate drug-disease associations predicted by MeSHOP similarity, we analyzed the 2007 baseline release of MEDLINE to generate predictions, and measured our predictive performance against annotations appearing in future releases of MEDLINE. The annual MEDLINE baseline releases 2007 and 2010 were used as the source of MeSH annotations for articles and were obtained directly from the NLM. The drug and disease MeSHOPs, computed for the MEDLINE baseline 2007, were compared using a panel of 16 similarity scores.

Future disease-drug relationships are predicted if MeSHOP comparison similarity scores exceed an applied threshold. Predictions were validated against drug-disease co-occurrences that appeared in the future MEDLINE releases which had not appeared in articles before 2007. A true positive novel association means an article referring to a previously unconnected drug-disease pair was published in the interim period between the 2007 and 2010 MEDLINE baselines.

As a second validation set, the Comparative Toxicogenomics Database (CTD) was used as a source of curated drug-disease relationships. We matched drugs from the 2011 CTD to the drugs defined in MEDLINE 2007, and defined a reference collection of 291 novel drug-disease relationships for those entries in CTD that were defined by publications appearing in the period of 2007-2011. The reference collection contains 191 unique drugs and 150 unique diseases.

Using these validation sets, we evaluate the candidate scoring methods by computing the Receiver Operating Characteristic (ROC) curve for predictions from analysis of the baseline 2007 data and reporting the Area Under the ROC Curve (AUC). Novel drug-disease pairs from the two reference sets are defined as "true positives", and all other drug-disease pairings are defined as "true negatives" (which is recognized to be conservative, as such pairs may be validated in future studies). All drug-disease pairs reported prior to 2007 are excluded from the AUC analysis.

The gold standard dataset analysed by the PREDICT algorithm [27] was mapped, with 574 of the 593 drugs mapping to 2007 MeSH pharmacologic compounds and the 190 of the 313 diseases mapping to MeSH Category C disease terms. A small number of drugs were not identified as pharmacologic compounds in 2007 MeSH. Diseases mapping to a combination of multiple MeSH disease phenotypes, or mapping to MeSH terms that were not in the Disease Category of MeSH were not included. Overall, 924 of the 1933 associations from the gold standard were mapped, comprising 406 drugs and 160 diseases. For the purposes of calculating the ROC validation curves, all other drug-disease associations between the mapped drugs and diseases are considered to be false positives. All the drug and disease mapped terms, as well as all the mapped gold standard drug-disease relationships are available for download at (http://meshop.oicr.on.ca/meshop/).

### Implementation

The analysis was performed using Python (http://www.python.org/), XSLT (http://www.w3.org/TR/xslt), and the MySQL database system (http://www.mysql.com/). Fisher's Exact Test p-values were computed using the R statistics package (http://www.r-project.org/). Results were generated using 50 CPUs of a compute cluster running under Sun GridEngine (http://www.oracle.com/technetwork/oem/grid-engine-166852.html). A typical cluster machine is a 64-bit dual processor 3 GHz Intel Xeon with 16 GB of RAM.

Data was leased and downloaded from MEDLINE/PubMed (http://www.nlm.nih.gov/databases/leased.html). The Comparative Toxicogenomics Database validation set was taken from the drug-disease relationships dataset (http://ctdbase.org/downloads/).

Results are freely accessible on the web at http://meshop.oicr.on.ca/meshop/. Source code implemented in Python is available at http://github.com/wac/meshop/ (drug and disease profile analysis) and http://github.com/wac/cmp-meshop/ (evaluation and validation of results).

## Results

### Generation of drug MeSH Over-representation Profiles (MeSHOPs)

**Figure 1:**
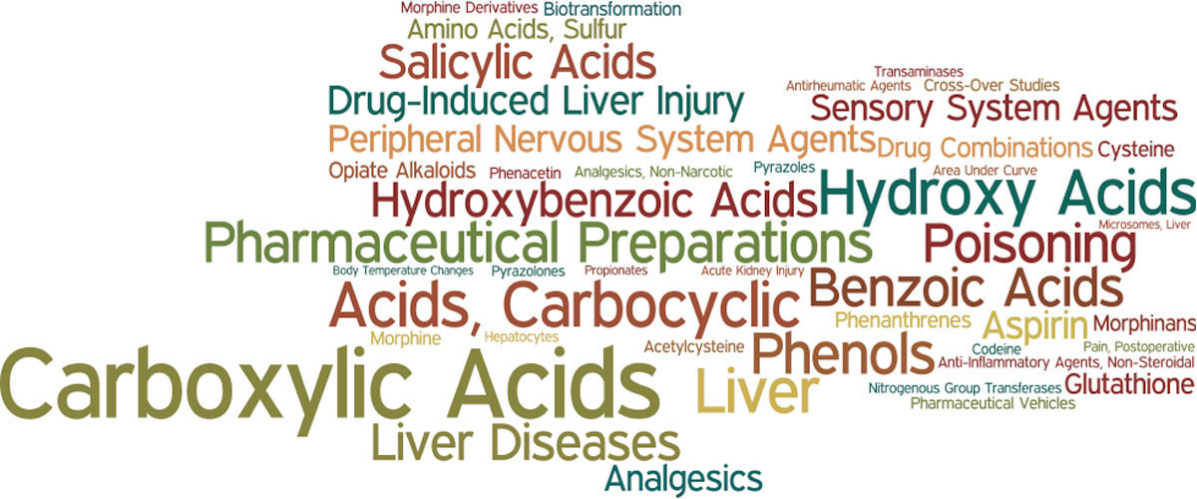
**MeSHOP for Acetaminophen**. All terms are presented in this MeSHOP word cloud associated in the Acetaminophen MeSHOP with a p-value of 0. The size of the term in the word cloud presented is proportional to the number of related articles for the term.

**Figure 2:**
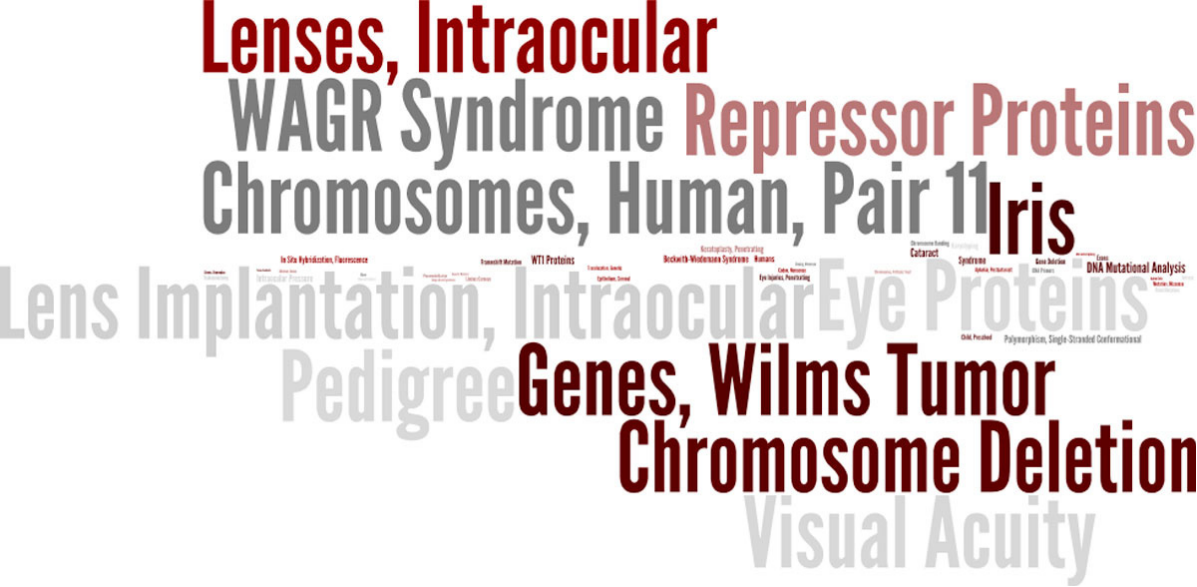
**MeSHOP for Aniridia**. The top 150 terms in the profile for the disease Aniridia are shown, where the font size of each MeSH term is proportional to the negative log p-value for the term.

MeSHOPs provide a quantitative overview of the biomedical knowledge associated with an entity of interest through the indexed biomedical terms. Following the described methods, MeSHOPs for all indexed diseases and drugs in MEDLINE were generated using archived MEDLINE data up until 2007. A drug MeSHOP is presented for acetaminophen (Figure 1), and a disease MeSHOP is presented for Aniridia (Figure 2). The scores within MeSHOPs are influenced by the background correction for the expectation of MeSH term frequency. If one takes the background rate from all articles in MEDLINE, MeSH terms preferentially associated with drugs are likely to be emphasized in the drug MeSHOPs, such as 'pharmaceutical preparation'. The strong scores for such drug-related terms can be corrected for by using class-specific backgrounds - such as the subset of articles that address one or more drugs. For comparisons of MeSHOPs across categories, as will follow, we select the universal background as a common background for all entities being compared.

### Predicting drug-disease associations

We examine the utility of drug-disease MeSHOP similarity scores for the prediction of drug-disease co-annotation in future publications. Table 2 demonstrates that comparison of drug and disease MeSHOPs predicts future drug-disease co-occurrence in subsequent years (2007-2011). The most effective similarity score is the Euclidean distance of log-p of overlapping terms only, which produces an AUC score of 0.95 for the prediction of future co-occurrence in publications:

**Table 2:** Performance of a selection of drug-disease similarity scores.

Scoring Method	Direct Connection Validation AUC	CTD Validation AUC	PREDICT Validation AUC
Corrected drug-disease p-value	0.65	0.76	0.66
Cosine distance tf-idf	0.88	0.91	**0.87**
Cosine distance of p-values	0.64	0.70	0.52
Cosine distance of term fractions	0.78	0.83	0.80
Sum of the log of combined p-values	0.92	**0.93**	0.80
Sum of the differences of log p values	0.89	0.86	0.58
L2 of log-p of intersecting terms	**0.95**	0.92	0.66
L2 of term fractions of intersecting terms only	0.64	0.55	0.57
L2 of log of p-values	0.88	0.84	0.57
L2 of p-values	0.87	0.82	0.56
L2 of term fractions P(s < S)	0.85	0.90	0.78
L2 of term frequency	0.87	0.83	0.62
Total number of terms	0.90	0.87	0.62
Number of Intersecting Terms	0.91	0.91	0.63
Number of Drug Terms	0.80	0.83	0.58
Number of Disease Terms	0.84	0.83	0.60

Performance validated using novel direct drug-disease direct co-occurrences from MEDLINE, and novel drug-disease relationships from the CTD. Top scores for each validation set are presented in boldface type.

$$\sqrt{\underset{ie\left(C\cap D\right)}{\sum }{\left(\text{log}\left(c_{p}\left(i\right)\right)-\text{log}\left(d_{p}\left(i\right)\right)\right)}^{2}}$$

(*C *and *D *refer to the MeSH terms of drug and disease MeSHOPs respectively, *c_p_(i) *and *d_p_(i) *refer to the p-value for the MeSH term *i *of the drug or disease profile respectively).

Enthusiasm for the performance is tempered, however, by the fact that a simple metric of the number of MeSH terms associated with a disease when used as a prediction ranking produces an AUC score of 0.84 (and counts for drug-associated MeSH terms produce a score of 0.80). Randomly assigned scores will produce an AUC of 0.5. These results are consistent with a process in which well-studied diseases (or drugs) are more likely to be the subject of future research publications and therefore more likely to co-occur with drugs than diseases that have few publications. These scores reflect a systematic limitation in the scoring procedure that needed to be resolved to allow for the identification of drugs suitable for orphan disorders, as well as to produce a more refined list of candidates to pursue.

When we examine the mapped validation evaluated by the PREDICT algorithm, we see a non-random but weaker predictive ability from the number of terms for the disease (AUC of 0.60) and the number of terms for the drug (AUC of 0.58).

Comparing drug-disease MeSHOP profiles can yield AUC of up to 0.87, comparing favorably to the AUC of 0.90 reported by the PREDICT algorithm on the unmapped gold standard dataset (See Table 2).

### Annotation bias observed for curated drug-disease relationships

**Figure 3:**
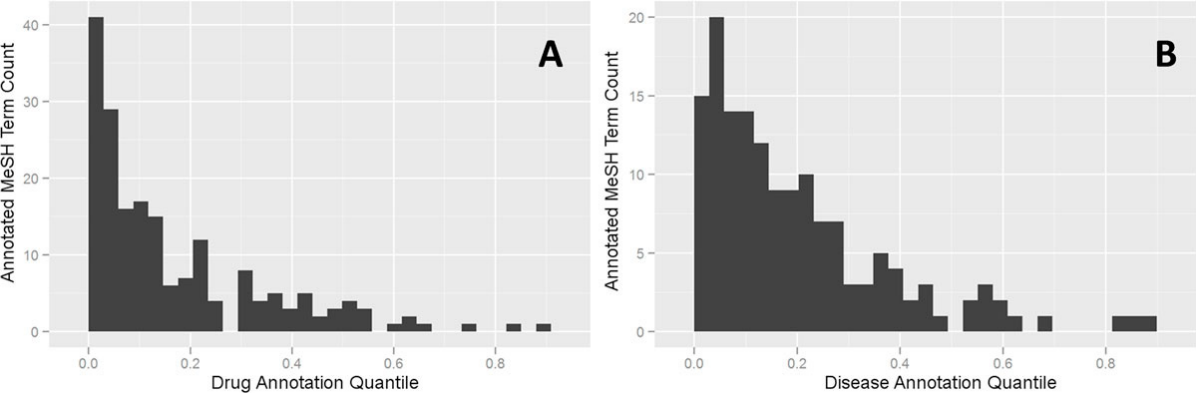
**Distribution of drug annotation (A) and disease annotation (B) in the new drug-disease associations of the CTD validation set**. The x-axis represents the quantile of the MeSH term counts for the drugs (part A) and diseases (part B) in the CTD reference collection (part A). The histograms indicate that both drugs and diseases within the CTD reference collection are biased toward greater numbers of associated MeSH terms.

Predicted novel drug-disease relationships were alternatively assessed against a curated reference collection from CTD that contains bonafide drug uses (i.e. not just co-occurrence in a paper, but manually assessed evidence that the drug is used as a treatment for a disorder). As seen in Table 2, similarity of MeSHOPs is able to accurately predict novel associations by comparing MeSHOPs of drugs and diseases, achieving ROC AUC of 0.93 (for the sum of the log of combined p-values). The Euclidean distance of overlapping terms metric that performed best for previous MeSHOP comparison performance tests, produces a similar ROC AUC of 0.92. As displayed in Figure 3, a substantial fraction of the validation set is over-represented for well-studied drugs and diseases. Over half of the 191 drugs are in the top 10% of all drugs in terms of amount of associated MeSH annotation (the peak to the left of the histogram). Only slightly less biased, of the 150 diseases, over half are in the top 15% of diseases, in terms of associated MeSH annotation. Consistent with these properties, using the baseline MeSH term counts for drug or disease annotation levels as scores, a ROC AUC of 0.83 is achieved. As for the co-occurrence measure, it is clear that annotation bias is a strong predictor for bona fide interactions.

### Controlling for annotation bias

**Figure 4:**
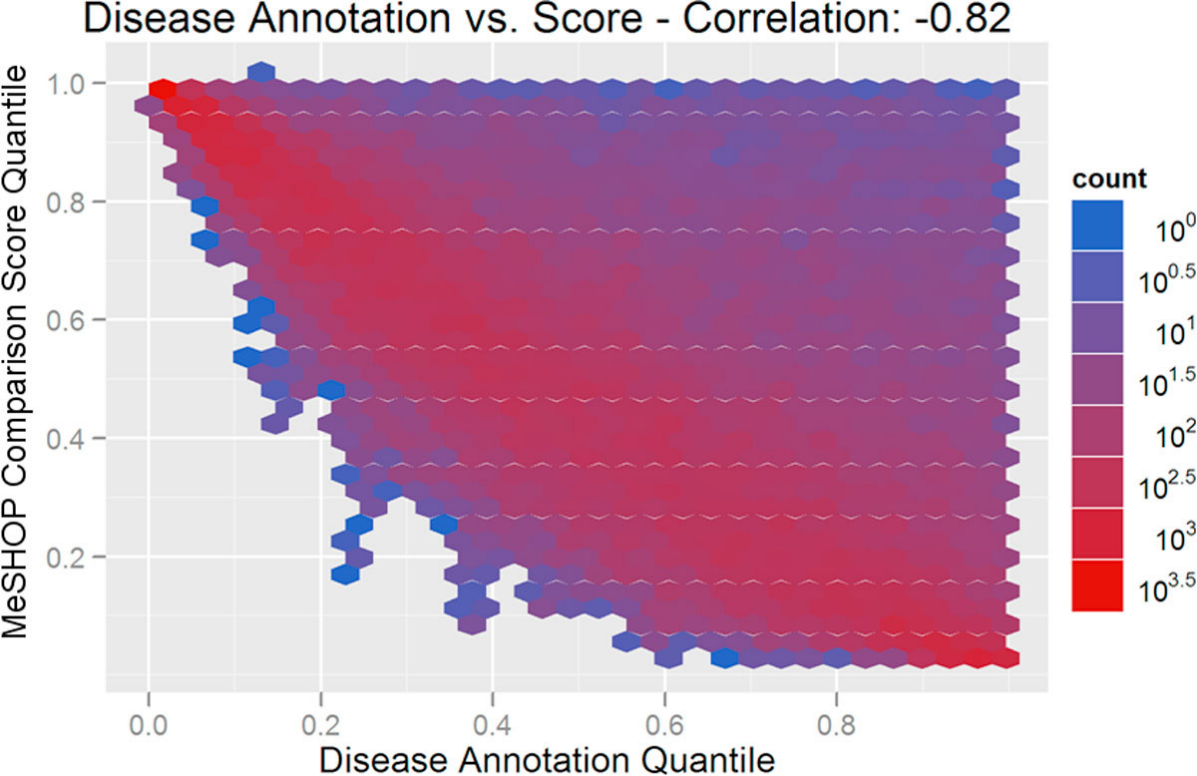
**The degree of disease annotation plotted against MeSHOP comparison score**. The figure displays a heatmap depicting the number of drug-disease tuples for a disease annotation level (MeSH terms attached to the disease MeSHOP) on the x-axis and a MeSHOP comparison score on the y-axis. MeSHOP similarity scores were calculated using Euclidean Distance. The degree of disease annotation, measured as the total number of distinct MeSH terms associated with a disease, is highly inversely correlated (Pearson correlation score of -0.82) with the similarity score.

**Figure 5:**
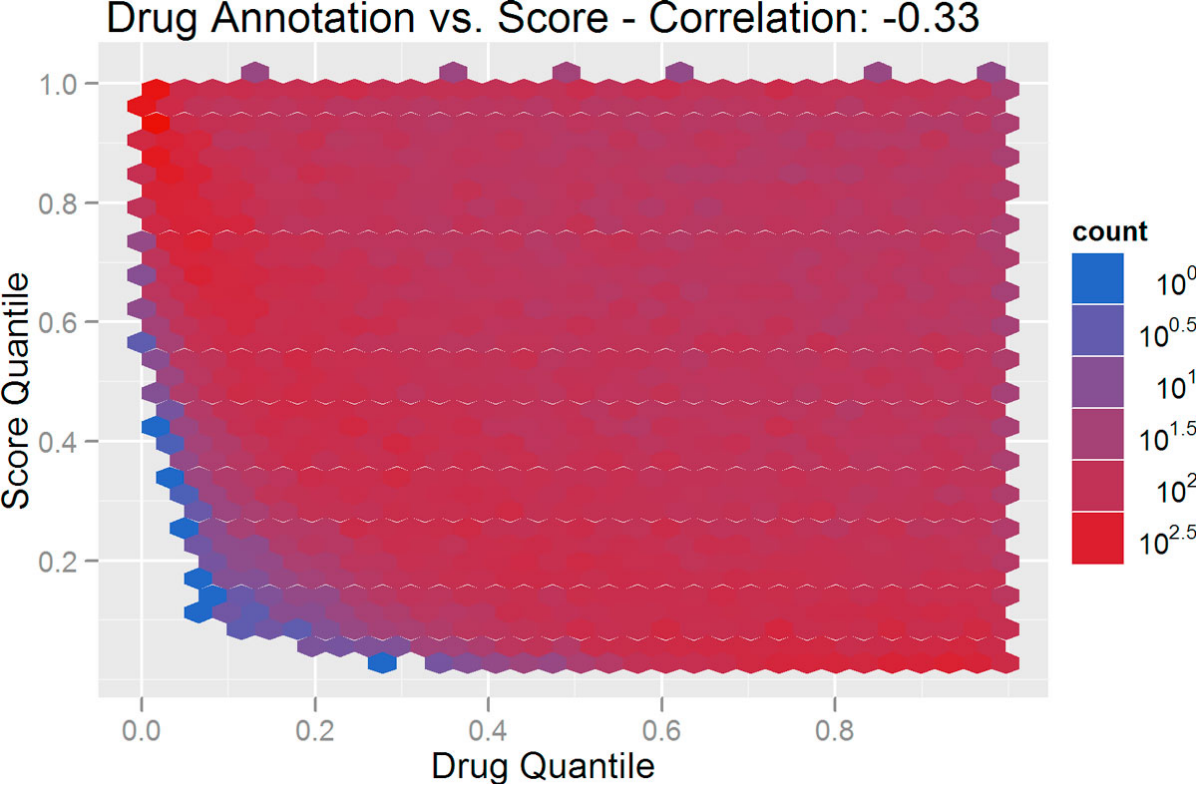
**The degree of drug annotation vs**. **MeSHOP comparison score**. The figure displays a heatmap depicting the number of drug-disease tuples for a drug annotation level (MeSH terms attached to the disease MeSHOP) on the x-axis and a MeSHOP comparison score on the y-axis. MeSHOP similarity scores were calculated using L2 distance. The degree of drug annotation, measured as the total number of distinct MeSH terms associated with a drug, is inversely correlated (Pearson correlation score of -0.33) with the similarity score.

**Figure 6:**
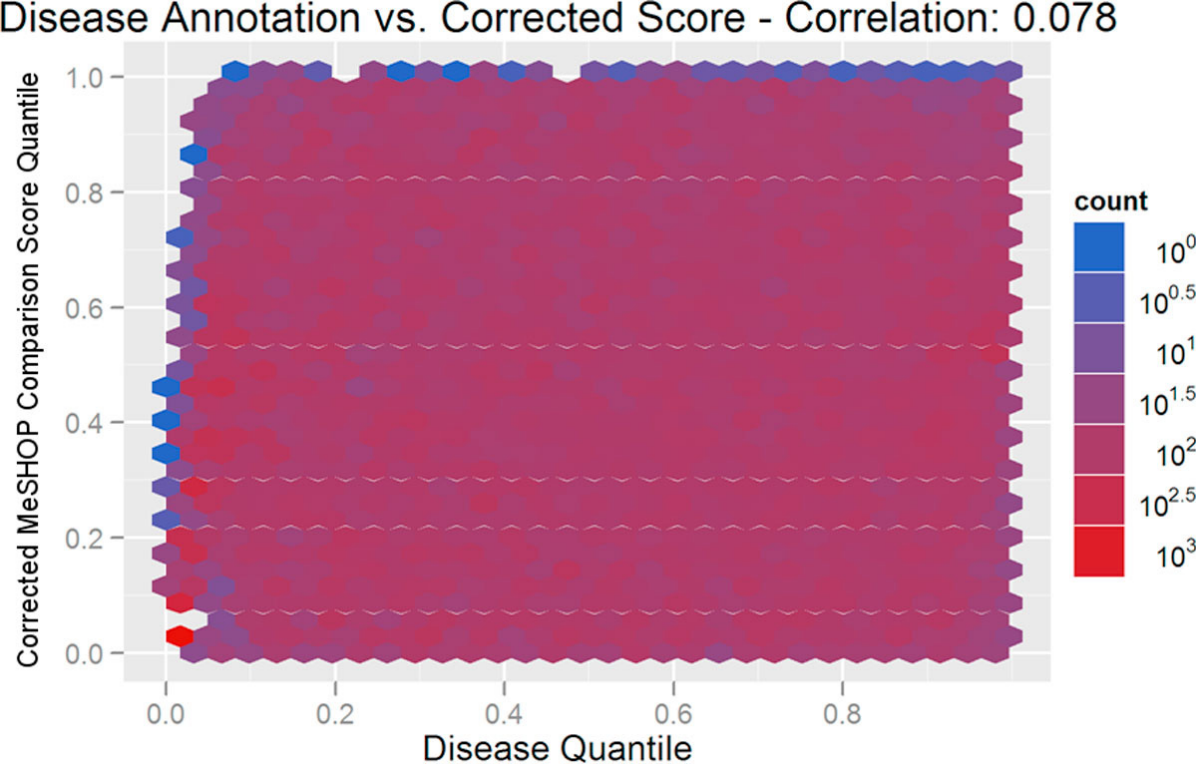
**Disease annotation vs**. **corrected MeSHOP comparison score**. The figure displays a heatmap depicting the number of drug-disease tuples for a disease annotation level (MeSH terms attached to the disease MeSHOP) on the x-axis and a corrected MeSHOP comparison score on the y-axis. MeSHOP similarity scores were calculated using L2 distance, but were corrected as described in the text to account for background annotation levels. The degree of disease annotation, measured as the total number of distinct MeSH terms associated with a disease, is no longer correlated (Pearson correlation score of 0.08) once corrected.

**Figure 7:**
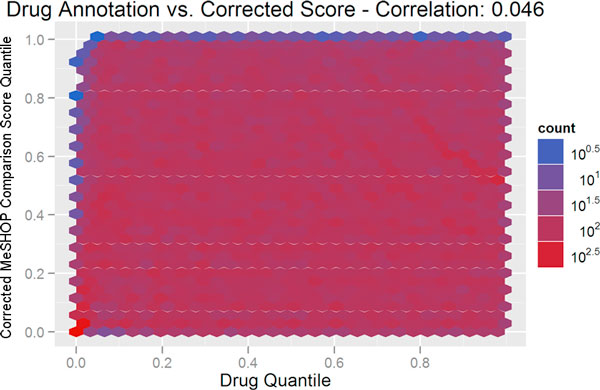
**Drug annotation vs**. **corrected MeSHOP comparison score**. The figure displays a heatmap depicting the number of drug-disease tuples for a drug annotation level (MeSH terms attached to the drug MeSHOP) on the x-axis and a corrected MeSHOP comparison score on the y-axis. MeSHOP similarity scores were calculated using L2 distance, but were corrected as described in the text to account for background annotation levels. The degree of drug annotation, measured as the total number of distinct MeSH terms associated with a drug, is no longer correlated (Pearson correlation score of 0.05) once corrected.

The influence of annotation on the MeSHOP comparison scores can be visualized using heatmaps. As seen in Figure 4, and fully consistent with the AUC scores above, there is a high degree of correlation between the amount of annotation for the disease (as measured by the number of MeSH terms in the disease profile), and the drug-disease score (Pearson correlation of -0.82). A correlation of -0.33 is observed when comparing drug-disease scores against the degree of drug annotation (see Figure 5). For a candidate list for drug repositioning, this annotation bias must be eliminated to allow for more rarely studied drugs or diseases to emerge from the analysis as candidates. We introduce a corrected scoring procedure for MeSHOP comparisons that computes the significance of similarity scores based on the distribution of scores for drug-disease tuples with similar annotation levels. In short, the observed similarity score should be remarkable given the level of annotation of the drug and disease in the tuple. After applying this correction for drug-disease annotation bias, both disease annotation level and drug annotation levels have very low correlation to the drug-disease score (0.08 and 0.05 respectively) (see Figure 6 and Figure 7). Table 3 demonstrates how the correction re-ranks the candidate drugs, shifting focus away from general compounds like monoclonal antibodies, immunoglobulin G, epinephrine and iron to compounds more directly to Arthritis and Gout. This also highlights some similar compounds that have not previously been linked to gout such as glucametacin and imidazole-2-hydroxybenzoate. We see similar results for the candidate drug lists for Asthma, Cardiac Arrhythmias, Jaundice and Lupus and provide the entire list of drug-disease relationships with raw and corrected scores online (See Additional file 1 and Supplementary Table 2 at http://meshop.oicr.on.ca/meshop/tbc2012.html).

**Table 3:** Comparison of top drug candidates for gout.

	Corrected Predictions			Original Predictions		
**Rank**	**Drug**	**Score**	**Articles**	**Drug**	**Score**	**Articles**

1	Kebuzone	0.14	2	Antibodies, Monoclonal	3.66E+08	4
2	Alminoprofen	0.17	1	Glucose	3.64E+08	5
3	Benziodarone	0.24	3	Insulin	3.24E+08	5
4	Proquazone	0.26	1	Norepinephrine	3.14E+08	1
5	Isoxicam	0.35	1	Tetradecanoylphorbol Acetate	3.02E+08	1
6	*Glucametacin*	*0.48*		Immunoglobulin G	2.98E+08	11
7	proglumetacin	0.52	2	Nitric Oxide	2.97E+08	5
8	*imidazole-2-hydroxybenzoate*	0.52		Interferon-gamma	2.88E+08	1
9	Prenazone	0.57	1	Serotonin	2.69E+08	4
10	*diclofenac hydroxyethylpyrrolidine*	*0.59*		Antibodies	2.62E+08	5

We compare the top 10 drug candidates after applying our described correction against the top 10 candidates before correction. Articles lists the number of MEDLINE articles in which the MeSH term "Gout" and the drug co-occur.

## Discussion

In this report, we introduce a new literature-based procedure for the analysis of drug-disease similarity with a focus on the identification of candidates for drug-repositioning. Using MeSH Over-representation Profiles (MeSHOPs) as quantitative representatives for biological entities, we seek to identify drugs and diseases with similar annotation under the expectation that such similarity may be suggestive of potential for repositioning. Drug-disease MeSHOP similarity scores, using a panel of metrics, are found to be strongly influenced by the level of annotation of drugs and diseases. The most heavily studied diseases and drugs are disproportionately emphasized by the comparison scores. A new corrected scoring procedure is introduced to account for the background expectation of similarity scores for comparably annotated drugs and diseases. The new procedure is demonstrated to account for the bias. Application of the MeSHOP similarity scoring procedure reveals a set of candidate drugs for future repositioning research.

The assessment of drug repositioning candidate predictions is necessarily problematic. Given the expense of validating drug efficacy, there is no reference collection against which to measure performance. In this report we elected to take as references two approaches. First, we predicted future co-occurrence in the research literature. This measure is indirect, as co-occurrence does not necessarily reflect a functional tie between the drug and disease. Furthermore, this measure is particularly susceptible to annotation influence - well studied drugs and diseases have a higher rate of future publications and are thus more likely to be linked. The second reference collection tested was extracted from the CTD, which records bonafide drug-disease links. The performance measurements reflect a similar literature bias on the CTD results, which may reflect a tendency for well-studied drugs to be tested for utility in well-studied disease therapy.

Within this report, we observe that the MeSHOP comparisons perform better than simple annotation measures, which indicates that the similarity assessment has value. Furthermore, we were able to identify and correct for the annotation bias influence on the analysis. It is our hope that future annotation-based similarity measures will be evaluated for the biases we observe here.

The source of the annotation biases identified in the validation sets may lie in methodological bias or be intrinsic to the nature of drug-disease relationships. The case for methodological bias notes the relationship between the existence of experimental protocols and the publication of related research. The study of disease involves the availability of appropriate animal models, a family with a history of the condition, a large-scale association study, and an accurate protocol to diagnose the condition. As well, the rarity and severity of the disease will also change the degree of research interest. Likewise, the study of drugs also benefits from animal models, bioassays to detect the compound, the ability and ease to generate the compound, and the ability to deliver an appropriate dosage of the compound to the targets of interest. Other factors motivating research directions are availability of funding and the focus of existing lab personnel and their research towards more popular directions of research.

However, the bias may also intrinsic to the nature of the disease or of the drug. Gillis and Pavlidis [28] have previously observed that multifunctional genes are a strong driver in gene function prediction. They identify gene multifunctionality through protein interaction and co-expression datasets, which encompass previous definitions of the "hub-ness" of a particular gene. A drug may have a more global effectiveness, due to targeting these multifunction genes or their pathways, and thereby be involved in more drug-disease associations. Similarly, there may be diseases that are involved in key processes, and therefore be the target of many potential drugs. Whether the biases are intrinsic to the biology of drugs and diseases, primarily introduced by the human nature in the research, or some combination of these factors will ultimately be revealed by the results of future research. As our knowledge of the nature of drugs and diseases increases and matures, the human elements and methodological biases will increasingly become less significant, leaving us to identify the degree this bias is due to the biological mechanism and nature of the drugs and diseases.

The underlying principle motivating the comparison approach to reveal novel drug repositioning candidates is that there will be shared characteristics of the drug actions and disease properties. While the current approach utilizes universal comparisons across all MeSH terms, it may be beneficial to restrict the analysis to a subset of more relevant MeSH terms. Development of a procedure to restrict the terms (the features) of MeSHOPs may allow for more specific drug repositioning candidates to emerge in the future.

### Future work

MeSH provides a wide spectrum of medically relevant topics, however, some applications may be better served by a vocabulary with more specific terms in the field of interest. For example, there are only eight terms in MeSH (Akathisia, Drug-Induced; Drug Eruptions; Drug Toxicity; Dyskinesia, Drug-Induced; Epidermal Necrolysis, Toxic; Erythema Nodosum; Serotonin Syndrome; Serum Sickness) relating directly to adverse drug events. Instead, there are several subheadings including "adverse effects", "poisoning", "toxicity" and "contraindications" which can occur with drug terms, or "chemically induced" and "complications" subheadings occurring with adverse outcomes. Expanding the analysis to look specifically for these subheading modifiers could allow us to extract a subset of articles directly relevant to adverse drug reactions for MeSHOP analysis. Alternatively, an alternative source linking side effects to articles could be employed to supplement our existing analysis with side-effect data.

CitationRank [29] was used to highlight genes involved in adverse drug reaction by analyzing the co-occurrence of genes in articles relating to an adverse drug reaction. Looking at the comprehensive network of MeSHOP similarity between genes, drugs and diseases would allow a similar network-style analysis, adding the information of the gene entities.

Rather than predicting drug-disease associations directly, another application of the method could be to highlight potential links between drugs and mechanisms of action. Drug therapies can be effective even when the understanding of the underlying mechanism of action is incomplete. These predicted drug-mechanism links could be also related back to relevant diseases, indirectly helping hypothesize on the biology of a disease and effective mechanisms for treatment.

## Conclusions

Comparing MeSHOPs allows quantitative analysis of MeSH biomedical topics shared between drugs and diseases through their MEDLINE-indexed primary literature. Quantitatively measuring MeSHOP similarity is shown to infer functional relationships between drugs and diseases. Specifically, the similarity between drug MeSHOPs and disease MeSHOPs is highly predictive of future drug-disease ties. The best similarity metric, using Euclidean distance of the log-p of overlapping terms, achieves a mean AUC of 0.94, an 11% improvement over baseline. However, bibliometric characteristics, such as the number of terms in the disease MeSHOP, are demonstrated to have a strong bias in drug-disease association. We describe here a correction that eliminates this bias in the scoring metrics, separating the effects of the similarity scoring from the annotation bias.

## Competing interests

The authors declare that they have no competing interests.

## Authors' contributions

All authors contributed to the design of the method and the analysis and interpretation of the data. WAC implemented and carried out the study. All authors read and approved the final manuscript.

## Funding

This work was supported by the Canadian Institutes for Health Research [to WWW]; the Ontario Institute for Cancer Research through funding by the government of Ontario [to BFFO]; the National Sciences and Engineering Research Council of Canada [to WWW and WAC]; the Michael Smith Foundation for Health Research (MSFHR) [to WWW and WAC]; the National Institute of General Medical Sciences [R01GM084875 to WWW]; and the Canadian Institutes of Health Research/MSFHR Strategic Training Program in Bioinformatics [to WAC].

## Acknowledgements

The authors are grateful to Leon French, Paul Pavlidis and Raf Podowski for comments and discussion on the research and Joseph Yamada for help with the website development.

## Declarations

The publication costs for this article were funded by the Centre for Molecular Medicine and Therapeutics (funds awarded to WWW) and the Ontario Institute for Cancer Research (funds awarded to BFFO).

This article has been published as part of *BMC Medical Genomics *Volume 6 Supplement 2, 2013: Selected articles from the Second Annual Translational Bioinformatics Conference (TBC 2012). The full contents of the supplement are available online at http://www.biomedcentral.com/bmcmedgenomics/supplements/6/S2.

## Supplementary Material

Additional file 1 **Comparison of drug-disease candidates for five disorders**. The top 20 drug candidates for gout, cardiac arrhythmia, lupus, jaundice and asthma are provided. We contrast the corrected and uncorrected drug candidate lists for each disorder. The uncorrected list is heavily biased to general compounds such as Monoclonal Antibodies, Norepinephrine and Iron, whereas the corrected drug candidates focus on drugs that are much more specific to the disorder. This file is in Excel format. Click here for file
